# Persistent human-associated microbial signatures in burial soils from the 17th and 18th century New York African burial ground

**DOI:** 10.1093/ismeco/ycaf181

**Published:** 2025-10-14

**Authors:** Carter K Clinton, Fatimah L C Jackson

**Affiliations:** Ancestry Soil Health and Evolutionary Studies (ASHES) Laboratory, North Carolina State University, 112 Derieux Pl., Raleigh, NC 27695, United States; Department of Biological Sciences, North Carolina State University, 112 Derieux Pl., Raleigh, NC 27695, United States; Human Analytics Division, QuadGrid Research Laboratory, 10009 Broad St., Bethesda, MD 20814, United States

**Keywords:** 16S rRNA gene sequencing, Qiime2, human microbiome, burial soil, historical metagenomics, African burial ground, microbial community succession, ancient DNA (aDNA), environmental microbial diversity

## Abstract

Understanding the long-term persistence of human-associated microbial signatures in burial soils offers a untapped insights into historical human health, decomposition, and ecological transformation. This study investigates whether centuries-old burial soils retain distinguishable microbial evidence of human decomposition using 16S rRNA gene sequencing on 81 samples from the New York African Burial Ground (NYABG), a 17th and 18th century cemetery for free and enslaved Africans. Comparative analyses against six control soils from nearby urban parks were conducted using QIIME2, ALDEx2, and ANCOM. Burial soils exhibited significantly greater alpha diversity (Faith’s PD, Shannon, observed ASVs; *P* < .01) and distinct beta diversity patterns (Bray-Curtis, UniFrac; PERMANOVA *P* = .001). Enrichment of *Firmicutes*, *Actinobacteriota*, and gut-associated genera such as *Bacillus* and *Ruminococcus* characterized burial soils, whereas oligotrophic taxa dominated controls. Tentative identifications of human-associated pathogenic genera (e.g. *Fusobacterium periodonticum*, *Prevotella pleuritidis*) were observed exclusively in burial soils, suggesting their origin from the interred individuals but requiring further validation. These findings demonstrate that soil microbiomes reflect host-associated microbial communities long after decomposition, providing a scalable, nondestructive approach for reconstructing ancient microbial communities and host-associated health signatures. This work establishes the NYABG burial soil microbiome as a valuable model for microbial archaeology and introduces a replicable framework for integrating environmental microbiology, bioarchaeology, and historical epidemiology through the lens of postmortem microbial ecology.

## Introduction

### Background and microbial significance

The human microbiome, a complex network of commensal, symbiotic, and pathogenic microorganisms, is integral to host metabolism, immunity, and disease processes [1]. While extensive research has characterized microbial community dynamics during life, the postmortem human microbiome remains underexplored, particularly in long-term burial environments. Following death, the microbial communities within and on the human body undergo rapid succession, initially dominated by endogenous gut microbiota before environmental taxa become more prevalent [2]. However, the extent to which human-associated microbial signatures persist in burial soil over centuries remains largely unknown [3, 4].

Soil microbial communities are highly diverse and shaped by complex biogeochemical interactions, but human decomposition can introduce a distinct ecological disturbance. The release of organic material, nitrogen, and microbial biomass from decomposing remains can alter soil pH, oxygen availability, and nutrient cycling, ultimately driving shifts in bacterial composition [5]. Studies suggest that gut-associated *Firmicutes* and *Proteobacteria* may persist in grave soil, where they are considered human-associated because they derive from the interred individual—whether through decomposition processes or as remnants of the person’s lifetime microbiota. The presence of more host-restricted genera (e.g. *Fusobacterium*, *Prevotella*) further strengthens the inference of a human origin [6]. These shifts have been well-documented in forensic taphonomy over short postmortem intervals (days to months), yet their long-term stability in archaeological burial sites spanning centuries has not been systematically investigated.

Investigating the microbial legacy of historical burials is crucial for advancing both forensic and microbial ecology research. Burial soil microbiomes may retain taxonomic and functional signatures of past human-associated microbial communities, offering a novel, nondestructive approach to studying historical populations. Such analyses are particularly valuable for sites where skeletal remains are inaccessible or ethically protected, allowing researchers to infer aspects of past human health, diet, and environmental exposures from burial soils alone.

### The New York African Burial Ground as a case study

The New York African Burial Ground (NYABG) is the oldest and largest known burial site of free and enslaved Africans in the United States, dating from ~1640 to 1797 [7]. Located in Lower Manhattan, the site contained an estimated 15 000 burials, of which 419 individuals were excavated before the area was designated a National Monument in 2006. The skeletal remains were extensively studied and later reinterred, leaving only burial soil samples available for future analyses.

The NYABG presents a unique opportunity to investigate the long-term persistence of human-associated microbial signatures in grave soil. The burials were excavated from depths ranging ~9–25 feet below the modern surface, with many graves sealed beneath urban infrastructure following 19th-century development [8]. This minimizes recent environmental disturbances, allowing microbial shifts attributable to human decomposition processes to be distinguished from surrounding soil ecology. Moreover, historical records provide contextual metadata, including inferred demographics, health conditions, and burial practices, which can be integrated with microbiome data to explore how microbial signatures reflect past human existence.

This study employs 16S rRNA sequencing (via Qiime2) to analyze the bacterial diversity of 81 NYABG burial soil samples and six New York City (NYC) control soils. Differential abundance analyses (ALDEx2 and ANCOM) were used to identify microbial taxa that significantly distinguish NYABG burial soil from environmental control samples. If burial soil microbiomes retain long-term human-associated microbial markers, these findings could establish a nondestructive metagenomic framework for reconstructing historical populations and postmortem microbial ecology. Initiated in 2015, this research represents one of the earliest systematic investigations of burial soil microbiomes serving as a foundational contribution to the field.

### Objectives of the study

The overarching goal of this study is to determine whether microbial signatures in burial soil retain evidence of long-term human decomposition and historical human-associated microbiomes. To achieve this, we analyze the bacterial community composition of historic burial soil from the NYABG using 16S rRNA gene sequencing on an Illumina MiSeq platform, followed by bioinformatic analysis with Qiime2, and compare it to local nonburial control soils from NYC to identify statistically significant microbial differences. Differential abundance analyses (ANCOM and ALDEx2) are employed to determine which taxa differentiate burial soil from environmental soil, potentially revealing microbial shifts associated with human decomposition processes.

Additionally, we assess the persistence of gut-associated bacterial taxa (e.g. *Firmicutes, Ruminococcus, Faecalibacterium,* and *Lactobacillus*) in NYABG soil samples to evaluate whether their presence serves as an indicator of past human presence and decomposition-driven microbial transformations. We also investigate the occurrence of human-associated pathogenic bacteria (e.g. *Salmonella, Prevotella, Legionella,* and *Fusobacterium*), which are linked to human hosts and have not been documented in environmental soil microbiomes. The detection of these taxa in NYABG burial soil suggests they were likely carried by the burial inhabitants, potentially reflecting infectious diseases that affected this historical population and may have contributed to causes of death.

Finally, this study aims to establish a nondestructive microbial metagenomics framework for investigating historical burial sites, offering an alternative approach to studying past populations when skeletal remains are unavailable or ethically protected. By addressing these objectives, this research contributes to microbial ecology, forensic taphonomy, and historical metagenomics, providing novel insights into long-term burial soil microbiomes, historical disease exposure, and their potential to reconstruct past human-associated microbial communities.

## Materials and Methods

### Sample collection

Soil samples from the NYABG were originally collected at the time of excavation in 1991 in direct association with human skeletal remains. A total of 81 burial soil samples were analyzed in this study, each labeled and categorized by burial number and the specific region of the body from which it was collected (e.g. Burial 310 cranial soil). These samples were extracted from within the crevices of skeletal remains, including regions such as the cranial cavity, thoracic cavity, and pelvic region, which likely contain remnants of decomposed human tissue and fine bone fragments ensuring that they represent microbial communities influenced by the decomposed burial inhabitant rather than surrounding plot soil. In this sense, burial soil represents a composite matrix of soil and decomposed remains, a metaphorical reflection of the phrase “ashes to ashes, dust to dust.” We therefore treat these samples with the same ethical considerations extended to human remains.

The depth of excavation ranged from ~9 to 25 feet below the modern surface, with many burials sealed beneath urban development following 19th-century construction. After collection in sterile clothbags, all samples were stored in temperature-controlled conditions (18–20°C) within steel cabinets, protected from ambient light, moisture, and contamination, which are known to preserve DNA integrity in soils for decades [9, 10], until the commencement of this genomic analysis in 2015. During processing, extraction blanks and negative controls were included at all steps to monitor for contamination from laboratory reagents or handling.

To compare NYABG burial soil with nonburial environmental soil, six control samples were collected from public parks within a 2-mile radius of the NYABG excavation site using a soil coring method. These sites included Washington Market Park (WMP), City Hall Park (CHP), Thomas Paine Park (TPP), Duane Park (DP), and Coleman Park (CP), along with a laboratory positive control (PC), mock community controls, and negative controls. Control samples were extracted from 40" to 48" below ground surface, ensuring that they represented soil minimally impacted by recent surface microbial activity. These control soils serve as nonburial, nonhuman-influenced environmental baselines against which the NYABG burial microbiomes were compared.

### DNA extraction and 16S rRNA gene sequencing

DNA was extracted from all soil samples using the Qiagen DNeasy PowerSoil Kit (Qiagen, Germantown, MD, USA), following the manufacturer’s protocol with modifications to optimize yield and quality given the age of the samples. Optimization included adding TE buffer to the soil and incubating at room temperature for 30 min, incubating the samples at 56°C before bead beating, and increasing the bead beating to two round of 10 min each to loosen particles and aid lysis. Vortexing durations were increased to 20 min, and centrifugation times were doubled at each step to maximize DNA recovery.

Sequencing libraries were prepared using V3-V4 region primers targeting the 16S rRNA gene, following the protocol described by Kozich *et al.* (2013) [11]. Amplicon sequencing was performed on an Illumina MiSeq platform using the MiSeq Reagent Kit v2 (500-cycle format) at the Center for Microbial Systems, University of Michigan. Paired-end reads were generated and demultiplexed using standard Illumina sequencing protocols.

### Bioinformatics and statistical analysis

Sequencing data were processed using Qiime2 (version 2022.2 and amplicon-2024.10) [12], a microbiome bioinformatics platform designed for multi-omics analysis. The raw paired-end 16S rRNA gene sequences were imported in Casava 1.8 demultiplexed format and converted into a Qiime2 artifact (.qza format) using the qiime tools import command. After import, sequences were subsampled (qiime demux subsample-paired with --p-fraction 0.3) and summarized using qiime demux summarize to evaluate sequence quality and distribution.

To ensure data quality, low-read samples (<100 reads) were filtered before denoising. DADA2 [13] was used for quality filtering, denoising, chimera removal, and paired-end read merging (qiime dada2 denoise-paired).

### Database justification

To assess the impact of reference database choice on taxonomic assignment, we compared ASV classifications generated using the SILVA 138 [14] and Greengenes 13_8 [15] databases. While there was perfect agreement at the Kingdom level (3160/3160 ASVs, 100%), concordance declined at finer taxonomic ranks: Phylum (47.6%), Class (51.2%), Order (34.4%), Family (56.0%), Genus (70.3%), and Species (0.0%). The apparent lack of species-level concordance was primarily attributable to divergent naming conventions, placeholder taxa (e.g. “uncultured”), and taxonomic reclassifications between databases.

Across all taxonomic ranks, 6.8% of SILVA assignments and 6.6% of Greengenes assignments were labeled as unclassified, uncultured, or otherwise unidentified, indicating that both databases left a similar proportion of ASVs without definitive taxonomic labels. However, SILVA consistently assigned more ASVs across all intermediate taxonomic ranks (Phylum through Genus) and incorporated recent phylogenetic updates such as *Actinobacteriota*, *Ruminiclostridium*, and *Candidatus Solibacter*. In contrast, Greengenes frequently returned legacy or ambiguous classifications (e.g. *da101*, *Oscillospira*). Based on its higher taxonomic depth, systematic rigor, and consistency with current microbial classification standards, SILVA was selected for all downstream analyses.

Sequences were cross-referenced against a curated list of bacterial pathogens compiled from NCBI RefSeq and published pathogen datasets to tentatively identify potential human-associated taxa. We emphasize that this pathogen database provides heuristic annotation and is limited by the short length of 16S amplicons.

### Diversity metrics

Alpha and beta diversity metrics were computed using qiime diversity core-metrics-phylogenetic, with a rarefaction depth of 800 to normalize read counts across samples. Alpha diversity metrics included Faith’s Phylogenetic Diversity, Shannon Diversity, Observed Features, and Evenness, while beta diversity was assessed using Bray–Curtis dissimilarity, weighted and unweighted UniFrac distances, visualized through PCoA plots (Emperor visualization).

A phylogenetic tree was constructed for diversity analysis using the qiime phylogeny align-to-tree-mafft-fasttree pipeline, which included multiple sequence alignment (MAFFT), masking of hypervariable regions, and tree generation with iTOL [16].

Taxonomic classification was performed using a Naïve Bayes classifier trained on the SILVA 138 database (qiime feature-classifier classify-sklearn). The taxonomic assignments were visualized using qiime taxa barplot.

Differential abundance analyses were conducted using ALDEx2 [17, 18] for compositional analysis and ANCOM [19] to identify significantly enriched taxa between NYABG and control soils. Phyloseq [20] in R (version 4.5.0) [21] was used for additional statistical analyses and visualization. All statistical analyses were performed in R with significance thresholds set at *P <* .05, and false discovery rate (FDR) correction applied to control for multiple comparisons.

## Results

### Sequencing depth and data quality

After quality filtering and denoising using DADA2 in the Qiime2 pipeline, a total of 68 samples from the initial 87, including 63 NYABG burial soil samples and five control soils were retained for downstream analysis. Samples with fewer than 1000 sequences were excluded based on rarefaction thresholding. The retained samples had sequence counts ranging from 1000 to 5169 reads, with a median depth of 1534 reads per sample.

Quality profiles generated by qiime demux summarize indicated consistently high per-base quality scores across both forward and reverse reads. Read truncation was performed at 240 bp (forward) and 220 bp (reverse), and primer sequences were removed prior to denoising. The DADA2 algorithm effectively filtered low-quality reads, removed chimeras, and merged paired-end reads, resulting in high-quality amplicon sequence variants (ASVs).

**Figure 1:**
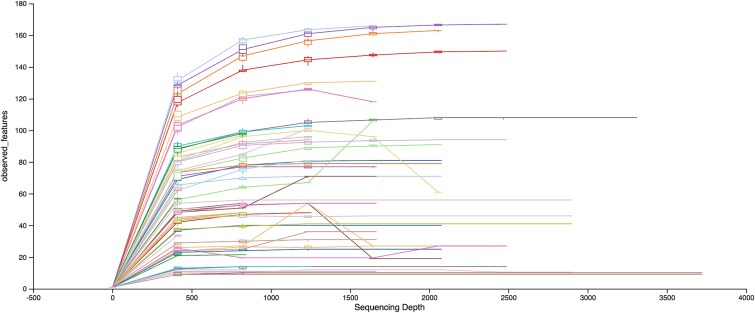
Rarefaction analysis of microbial richness in NYABG burial and control soils. Rarefaction curves show observed ASVs as a function of sequencing depth for all samples. Burial soils from the New York African Burial Ground (NYABG) consistently show higher richness at all sequencing depths compared to control soils (1000 reads), indicating sufficient coverage for diversity analyses.

Rarefaction curves demonstrated that most samples reached an asymptote in observed richness near the rarefaction depth of 1000 reads, indicating that this depth was sufficient for robust alpha and beta diversity estimation (Fig. 1).

### Taxonomic composition of burial vs. control soils

**Figure 2:**
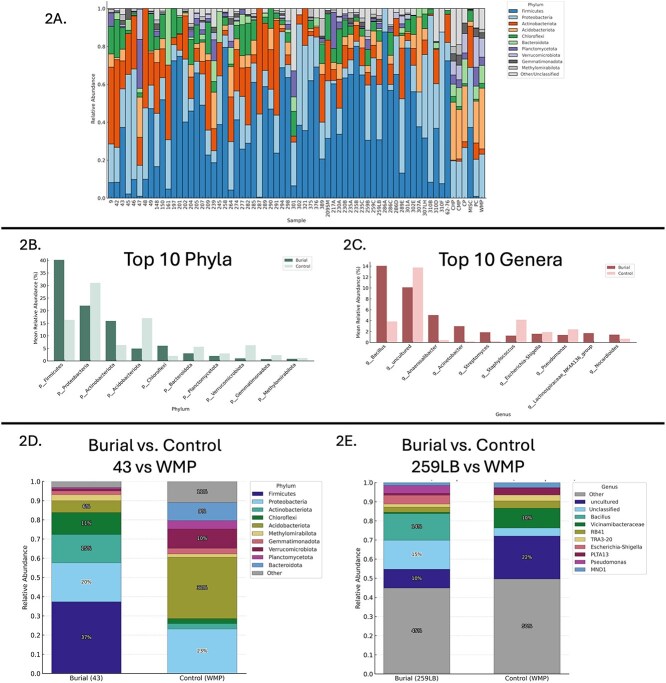
A–E. Taxonomic composition of microbial communities in burial and control soils. (A) Relative abundance of dominant bacterial phyla across all samples. (B) Phylum-level comparison between NYABG burial soils and controls, with significant differences indicated (*p* < .01). (C) Genus-level relative abundance profiles highlighting enrichment of *Bacillus*, *Anaerosalibacter*, and *Acinetobacter* in burial soils. (D and E) Manhattan distance-based clustering illustrates consistent taxonomic separation between representative burial and control soil samples.

Taxonomic classification of 16S rRNA gene sequences, revealed clear compositional differences between burial and control soils at multiple taxonomic levels, highlighting distinct ecological signatures associated with each group. Taxonomic classification across samples at the phylum level can be seen in Fig. 2A.

At the phylum level, burial soils were dominated by *Firmicutes*, which constituted ~40% of the average relative abundance, followed by *Proteobacteria* (22%) and *Actinobacteriota* (16%). In contrast, control samples were enriched in *Proteobacteria* as the most abundant phylum (31%), followed by *Chloroflexi* (17%), and *Firmicutes* (~16%) (Fig. 2B). Additional phyla such as *Acidobacteriota*, *Bacteroidota*, and *Planctomycetota* were also more prevalent in controls.

At the genus level, burial samples were enriched in *Bacillus* (~14%), uncultured *Firmicutes* (10%), and *Anaerosalibacter* (~5%), as well as *Acinetobacter* (~3%). By contrast, control soils exhibited higher relative abundance of uncultured *Proteobacteria* (~14%), *Escherichia-Shigella* (~4%), and *Pseudomonas* (~3%), reflecting more typical environmental microbial communities (Fig. 2C). These data highlight a divergence in dominant genera between the two groups.

To validate these distinctions, representative samples were selected for each group using a Manhattan-distance–based similarity index. Because taxonomic distributions vary by level, different samples were selected as representatives for phylum (Burial 43 vs. WMP), family (259LB vs. WMP), and genus (259LB vs. WMP) comparisons. Representative comparisons at each level consistently reinforced the taxonomic divergence between burial and control samples (Fig. 2D and E).

Control soils exhibited a consistently higher proportion of unclassified and uncultured taxa across taxonomic levels, indicating greater microbial novelty. At the phylum level, unclassified taxa were more abundant in controls (8%) than in burial soils (2%), with no uncultured phyla detected. This trend intensified at the genus level, where uncultured taxa comprised 23% of control communities versus 10% in burial soils, and unclassified taxa accounted for 12% versus 7%, respectively. These patterns suggest that burial soils harbor more confidently annotated, human-associated taxa aligning with well-characterized reference genomes, while control soils reflect the complexity of native, less-characterized microbial diversity.

### Microbial diversity analyses

#### Alpha diversity

Burial soils from the NYABG exhibited significantly greater alpha diversity compared to nearby environmental controls across multiple metrics. Faith’s Phylogenetic Diversity (PD), Shannon Index, and Observed ASVs were all elevated in burial samples (*p =* .001, .001, and .003, respectively), indicating broader microbial richness and phylogenetic depth. Pielou’s Evenness did not differ significantly between groups (*p =* .097), suggesting comparable distribution of relative abundances.

**Figure 3:**
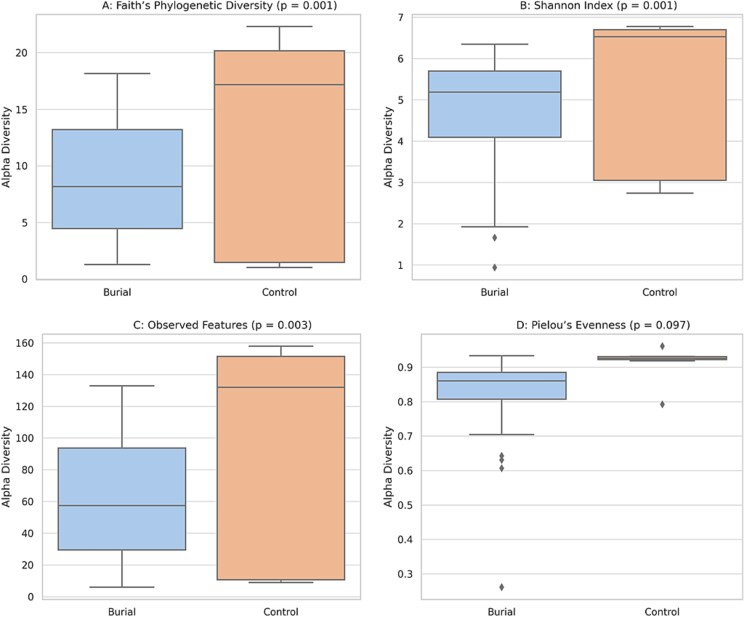
A–C. (A) Burial soil samples from the New York African burial ground (*n* = 63) exhibited significantly higher phylogenetic diversity than NYC environmental control soils (*n* = 5), as measured by Faith’s PD (*p* = .001). This suggests a broader evolutionary span of microbial lineages in burial-associated soils. (B) The Shannon diversity index was significantly higher in burial soils compared to controls (*p* = .001), indicating greater taxonomic richness and evenness in microbial community composition within the burial environment. (C) Burial samples had a significantly greater number of observed amplicon sequence variants (ASVs) than control soils (*p* = .003), suggesting elevated microbial richness associated with long-term human decomposition. (D) Pielou’s Evenness showed no significant difference between burial and controls (*p* = .097), indicating comparable community evenness.

These findings were supported by pairwise Kruskal-Wallis tests and confirm that NYABG burial soils host richer and more phylogenetically diverse microbial communities than nearby control soils (All Burials vs. All Controls: *p* = .003). The diversity enhancement is consistent with the long-term influence of human decomposition and nutrient influx (Fig. 3A–D).

#### Beta diversity

Beta diversity analysis revealed statistically significant differences in microbial community composition between NYABG burial soils and environmental controls across all tested distance metrics. PERMANOVA tests showed robust group separation for Bray–Curtis dissimilarity (pseudo-F = 2.639, *p =* .001), Unweighted UniFrac (pseudo-F = 3.034, *p =* .001), and Weighted UniFrac (pseudo-F = 5.272, *p =* .001) indicating compositional and phylogenetic differences in both presence/absence and abundance-weighted dimensions.

**Figure 4:**
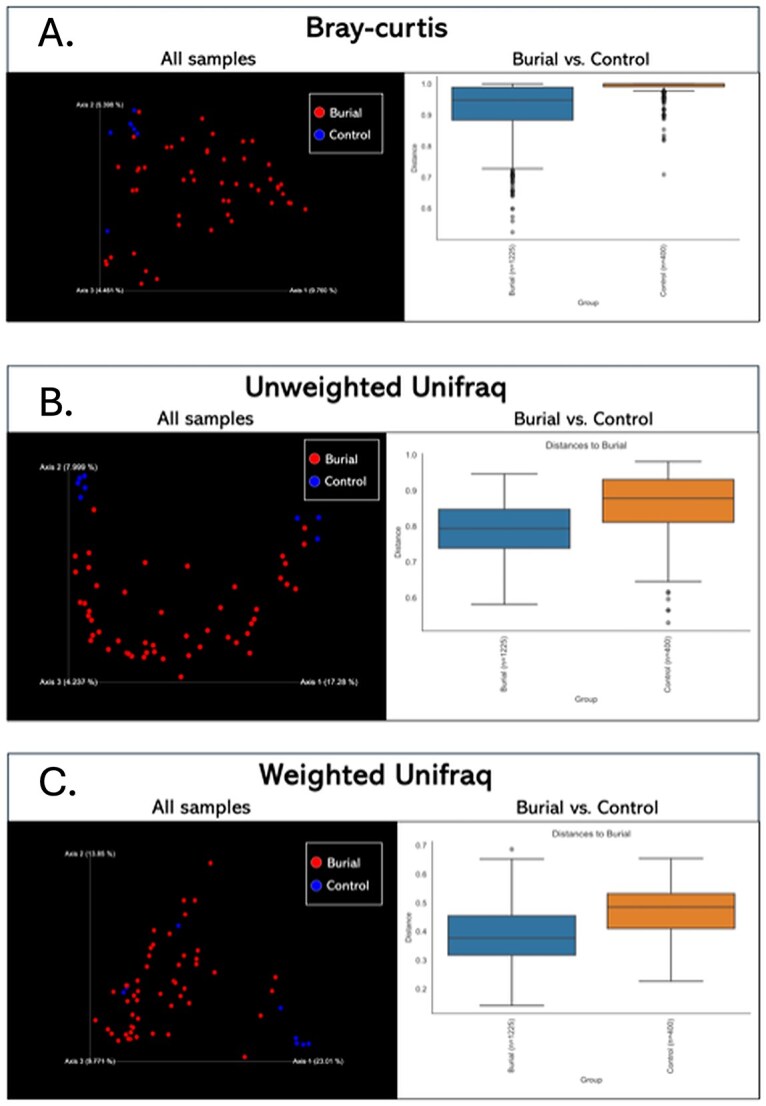
A–C. Beta diversity analyses show significant differences in microbial community composition between NYABG burial and control soils across taxonomic and phylogenetic metrics. Each panel shows a Principal Coordinates Analysis (PCoA) plot (left) of microbial communities based on a given distance metric, alongside a corresponding boxplot (right) representing pairwise distances used for group significance testing between burial and control soils. (A) Bray–Curtis dissimilarity highlights compositional differences between burial and control samples. Burial soils cluster tightly and separately from controls, with significant group differences confirmed by PERMANOVA (pseudo-F = 2.639, *p* = .001). (B) Unweighted UniFrac captures phylogenetic differences based on presence/absence of taxa. Clear separation is observed between burial and control groups (pseudo-F = 3.034, *p* = .001). (C) Weighted UniFrac integrates both phylogeny and abundance, showing the greatest group separation (pseudo-F = 5.272, *p* = .001), indicating that both lineage identity and relative abundance contribute to divergence.

Principal Coordinates Analysis (PCoA) plots corroborate these findings, revealing strong clustering of NYABG burial samples and greater dispersion among control samples. The greatest separation was observed with the Weighted UniFrac metric, suggesting that differences in both microbial phylogenetic composition and taxon abundance drive the divergence between burial and control groups. In contrast, Unweighted UniFrac captures differences based solely on phylogenetic presence/absence, and Bray-Curtis reflects differences in taxonomic abundance without phylogenetic context (Fig. 4A–C).

PERMDISP tests indicated no significant differences in group dispersion across all metrics (*p* > .68), confirming that observed compositional divergence is attributable to true ecological differences rather than variance artifacts.

### Differential abundance

#### Analysis of composition of microbiomes

**Figure 5:**
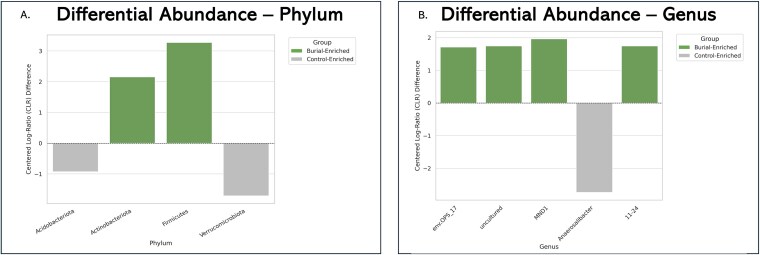
A and B. Differential abundance of bacterial phyla between NYABG burial and control soils. (A) Bar plot showing centered log-ratio (CLR) transformed differences in relative abundance for four phyla identified by ANCOM as significantly different between groups. *Firmicutes* and *Actinobacteriota* were enriched in burial soils, while *Acidobacteriota* and *Verrucomicrobiota* were more abundant in control soils. Positive CLR values indicate higher relative abundance in burial soils; negative values indicate enrichment in control soils. (B) Bar plot displaying the five genera with the highest ANCOM W-statistics and statistically significant CLR-transformed differences in abundance between groups. Genera such as *MND1* and *env.OPS_17* were enriched in burial soils, while *Anaerosalibacter* was significantly more abundant in control soils.

Analysis of Composition of Microbiomes (ANCOM) was used to identify differentially abundant taxa between burial and control soil samples. It identified 18 taxa with significantly different abundances between burial and control soils. At the phylum level, two phyla were significantly more abundant in burial soils: *Firmicutes* (W = 43) and *Actinobacteriota* (W = 41). In contrast, control soils exhibited higher abundance of *Verrucomicrobiota* (21) and *Acidobacteriota* (W = 3), consistent with native environmental communities. (Fig. 5.).

At the genus level, taxa enriched in control soils included *Anaerosalibacter* (*Firmicutes*), uncultured *Pedosphaeraceae* (*Verrucomicrobiota*), and *Subgroup* 7 (*Acidobacteriota*), consistent with microbial communities adapted to oligotrophic, plant-associated environments. In contrast, members of the *Microcillaceae* and *Chitinophagaceae* families, along with *MND1* (*Gammaproteobacteria*), were enriched in burial soils and are often associated with anaerobic degradation, spore formation, or anthropogenic enrichment(Fig. 5B).

#### ALDEx2

We conducted a differential abundance analysis using ALDEx2, focusing on taxonomic variation across samples from different locations. ALDEx2 analysis did not yield statistically significant ASVs (we.eBH < 0.05) after FDR correction; however, several taxa displayed large effect sizes suggestive of meaningful biological differences. These findings provide complementary support to ANCOM results and enhance resolution at finer taxonomic scales.

**Figure 6:**
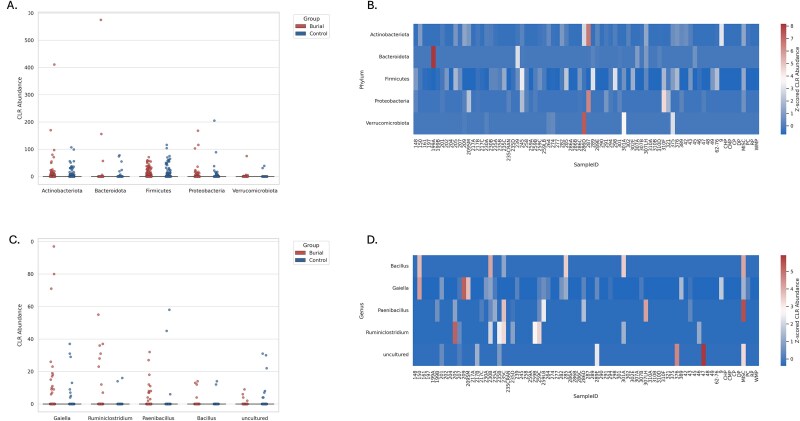
A–D. Boxplot of CLR-transformed phylum-level abundances comparing burial and control soils. (A) Top five phyla with the largest ALDEx2 effect sizes are shown. Burial soils show increased abundance of *Firmicutes* and *Actinobacteriota*, while *Verrucomicrobiota* and *Bacteroidota* are more abundant in control soils. Points represent individual samples. (B) Top five genera identified by ALDEx2 as having the highest absolute effect sizes are shown. Points represent individual samples, and groups are colored red (burial) and blue (control). Notable taxa enriched in burial soils include *Ruminiclostridium* and *Paenibacillus*, while uncultured genera were more prominent in controls. (C) Phylum-level heatmap patterns of differential abundance mirror genus-level trends. Burial-associated phyla such as *Firmicutes* and *Actinobacteriota* exhibit distinct clustering from environmental controls. (D) Heatmap of genus-level CLR abundance (z-scored) across samples displays the relative abundance across burial and control samples. Samples are ordered horizontally, and genera are clustered by row.

At the phylum level *Actinobacteriota*, *Bacteroidota*, and *Firmicutes* demonstrated greater CLR-transformed abundance in burial soils (Fig. 6A and B). At the genus level, *Bacillus, Paenibacillus, and Ruminiclostridium* were consistently elevated in burials, while controls harbored higher levels of *Stenotrophomonas*, *Sphingomonas*, and uncultured *Acidobacteria* (Fig. 6C and D).

Species-level resolution was also assessed, though a large proportion of ASVs remained unclassified or labeled as “uncultured” at this level. After excluding uncultured taxa, the most abundant identifiable species included *B. licheniformis, Hydrogenispora ethanolica*, and *P. polymyxa*.

Overall, the overlap between ALDEx2 and ANCOM reinforces the presence of a stable set of human-associated and decomposition-enriched taxa in burial soils, supporting their use as microbial indicators of long-term postmortem transformation.

#### Detection of human-associated microbial signatures

To evaluate whether burial soils preserve long-term microbial indicators of the human host, we conducted a targeted screen for human-associated bacterial taxa. We generated a curated database of human-associated microbiota, including gut, skin, oral, and urogenital commensals, as well as obligate and opportunistic pathogens. This database, generated using Open AI-4.0 GPT [22] API pulled from the NIH Human Microbiome Project [23], CDC infectious disease repositories [24], and published literature, was used to screen taxonomic assignments derived from 16S rRNA sequencing of burial soil.

To reduce the likelihood of false positives or environmental contaminants, we applied a conservative abundance threshold: only taxa with >20 reads per burial sample were considered biologically meaningful. All human-associated microbiome taxa described below exceeded this threshold and were consistently detected across the majority of burial samples, whereas their abundance in control soils was negligible (<5 reads) or absent.

We detected multiple bacterial taxa that are rare or absent in soil but well-established members of the human microbiome, supporting their introduction via decomposition. We also identified multiple pathogenic species highly specific to humans, many of which are not environmentally ubiquitous. Table 1 summarizes detected human-associated taxa meeting read-count and specificity thresholds. These organisms are composed of gut commensals and opportunistic pathogens.

**Table 1:** Detection of human-associated commensal and pathogenic bacterial taxa in NYABG burial soils.

Taxon	Category	Known host association	Function, significance, or association	Burial detection	Reference(s)
Detected human-associated commensal taxa					
*A. muciniphila*	Commensal	Gastrointestinal tract, gut mucosa, colon, cecum, small intestine, pancreas	Mucin-degrading gut symbiont; promising next-generation probiotic candidate	274, 286D, 301A, 375	[52]
*B. ovatus*	Commensal	Gastrointestinal tract, colon, female genital tract	Mucosal immunity, plays a role in SCFA^1^ production	239	[53, 54]
*B. vulgatus*	Commensal	Gastrointestinal tract, colon	SCFA^1^ production, metabolic regulation	245, 307A	[53, 54]
*B. uniformis*	Commensal	Gastrointestinal tract, colon	SCFA^1^ production, immune modulation	289	[53, 54]
*L. murinus*	Commensal	Digestive tract, oral cavity	Common member of the gut microbiome, a potential probiotic; plays a role in oral health by modulating inflammation	289E, 301, 301A, 375	[55, 56]
*Parabacteroides goldsteinii*	Commensal	Human colon	Anti-inflammatory effects, obesity modulation	245	[57]
*R. gauvreauii*	Commensal	Gastrointestinal tract	Isolated from human fecal sample, present in human bile	375	[58]
Detected human-associated pathogenic taxa					
*C. kroppenstedtii*	Pathogen	Female breast tissue	Granulomatous mastitis, breast infections	245	[59]
*C. tuberculostearicum*	Pathogen	Skin, vagina	Opportunistic infections in immunocompromised patients	245, 274, 286D, 287, 307LH, 375	[60, 61]
*F. periodonticum*	Pathogen	Oral cavity	Periodontal disease, oral carcinoma	259B, 310D	[62]
*P. melaninogenica*	Pathogen	Oral/respiratory tract	Chronic lung disease, aspiration pneumonia,	199A, 307LH, 45	[63, 64]
*P. pleuritidis*	Pathogen	Respiratory tract	Pleural infections; emerging respiratory pathogen	307A	[65]

*This table summarizes bacterial taxa identified in burial soils that are known to be associated with the human microbiome or pathology. Commensal taxa include members of the gastrointestinal and mucosal microbiota with documented roles in host*

*metabolism, immunity, and gut homeostasis. Pathogenic taxa consist of opportunistic and obligate pathogens associated with oral, dermal, respiratory, and urogenital infections. Detection was limited to burial samples in which taxa exceeded a conservative abundance threshold (>20 reads), minimizing spurious assignments. Each entry includes known host association, functional relevance, specific burials in which the taxon was detected, and supporting references. SCFA, short-chain fatty acids.*

^1^SCFA: short-chain fatty acids

#### Phylogenetic structure of dominant taxa

**Figure 7:**
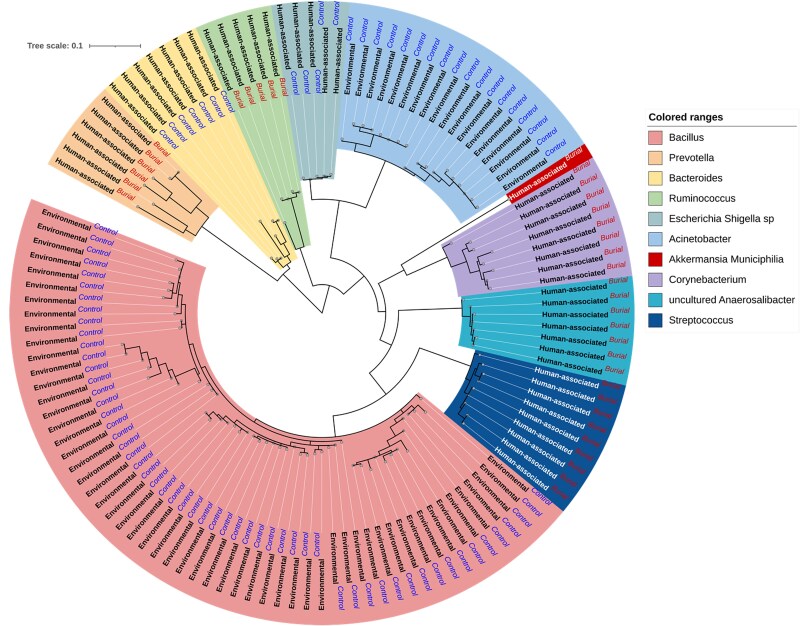
Phylogenetic tree of the top 10 most abundant bacterial genera across burial and control soils. A maximum-likelihood tree was generated from amplicon sequence variants (ASVs) corresponding to the 10 most abundant bacterial genera across all samples. Tip labels are annotated with ecological origin (human-associated or environmental) and enrichment group (burial-enriched or control-enriched). Clades are colored by genus-level taxonomy.

To investigate evolutionary relationships and ecological origins among dominant taxa, a maximum-likelihood phylogenetic tree was constructed using ASVs from the 10 most abundant bacterial genera across all samples (Fig. 7). Tip labels were annotated with both source origin (human-associated vs. environmental) and group-level differential abundance (burial-enriched vs. control-enriched).

Distinct clades reflected strong taxonomic coherence by genus, with burial-enriched ASVs predominantly clustering within human-associated taxa, including *Streptococcus*, *Corynebacterium*, *Akkermansia*, and *Escherichia-Shigella*. These taxa formed tight phylogenetic clusters, suggesting ecological and evolutionary filtering in the burial environment. In contrast, control-enriched ASVs, largely from genera such as *Bacillus*, *Prevotella*, and *Ruminococcus*, exhibited more phylogenetically dispersed patterns, consistent with their adaptation to diverse environmental niches.

The dual overlay of enrichment group and ecological origin revealed that burial soils disproportionately retain lineages taxonomically affiliated with human hosts, while control soils reflect broader environmental diversity.

#### Ancient DNA authentication

To confirm that burial soil microbiomes might include authentic ancient microbial DNA rather than modern contaminants, we conducted postmortem DNA damage analysis using DamageProfiler [25]. This tool, selected for its compatibility with short amplicon reads, was used in place of mapDamage2 [26], which is standardly used. The16S reads were aligned to a representative *B. subtilis* genome (NCBI RefSeq: GCF_000009045.1), and terminal C → T and G → A misincorporation frequencies were quantified.

Across most burial and control samples, no substantial damage signal was detected, consistent with expectations for PCR-amplified 16S data. However, one burial sample, B150 (150_S12_L001), exhibited low-level terminal deamination (C → T: 0.49%; G → A: 0.24%), suggesting limited preservation of damaged microbial DNA in select contexts or that the recovered microbial DNA largely reflects dormant or metabolically quiescent microbes, which replicate slowly or not at all in soil environments [9]. While some recovered sequences may represent near-extant microbial outgrowth within the burial environment or due to storage conditions, 16S rRNA sequencing is not the most adequate method for assessing ancient DNA authenticity. To address this limitation, we have performed shotgun metagenomic sequencing on these samples in a follow-up study, which will allow more definitive evaluation of ancient microbial DNA. Overall, given the metabolic activity of soil systems and the limitations of amplicon-based resolution, this analysis was not definitive and is included for methodological completeness.

## Discussion

### Persistence of microbial signatures in burial soils

This study establishes that biologically meaningful microbial signatures derived from human decomposition can persist in burial soils for centuries. Through 16S rRNA gene amplicon sequencing of 74 samples from the NYABG, we show that host-derived taxa remain detectable long after interment, distinguishing burial soils from nearby environmental controls both taxonomically and functionally. These findings extend the temporal window of microbial forensic detection and provide a nondestructive method for reconstructing aspects of the human past. Unlike skeletal remains, which are often inaccessible due to legal, ethical, or conservation concerns, burial soils serve as a biologically rich resource for ancient microbiome analysis.

### Microbial ecology and archaeometagenomic implications

Our results corroborate and extend prior research in microbial succession, which has focused largely on early postmortem intervals [27]. While previous work emphasized decomposition stages over days to months, our study demonstrates the persistence of microbial community signatures in soil for centuries. Burial soils exhibited increased alpha diversity and distinct beta diversity compared to control soils, with consistent enrichment of *Firmicutes* and *Actinobacteriota*, and human-associated genera such as *Bacillus*, *Anaerosalibacter*, *Bacteroides*, and *Ruminiclostridium*. These results align with recent findings that decomposition enriches soil microbiota through both nutrient influx and microbial dispersal from the body [28], and they suggest that certain host-derived taxa may become stable components of the soil microbial community over time.

This enrichment suggests burial environments act as selective microhabitats that preserve specialized taxa over time. Our results align with ecological theories of succession, showing that an early bloom of gut-associated microbes can stabilize into a decomposition-resistant microbial community [29–31]. Such findings support the concept of microbial legacy effects, whereby transient biological inputs exert lasting influence on environmental microbial communities [32].

### Human-associated, commensal, and pathogenic taxa

By integrating a custom-curated reference of human-associated taxa with our annotated 16S dataset, we identified multiple obligate gut commensals and opportunistic pathogens with predominant associations with the human body in burial samples. These include *R. gnavus*, *B. ovatus*, and *A. muciniphila*, all strongly host-associated and functionally linked to gut health, metabolism, and immunity [33]. We also detected taxa such as *F. periodonticum*, *P. melaninogenica*, and *C. kroppenstedtii*, which are rarely found in soil and associated with oral, respiratory, and breast infections [34–36]. Notably, these taxa not identified in control samples, suggesting that they may reflect individual variation in health or potential cause of death, reinforcing the biological specificity of these signals. The tentative identification of human-associated genera is intriguing but must be interpreted cautiously, as these assignments are based on 16S fragments and heuristic database matching. These results are best regarded as hypotheses that warrant verification through shotgun metagenomic analyses or targeted qPCR.

### Phylogenetic insights into human decomposition

Phylogenetic analysis of dominant genera further reveals that burial-enriched taxa, including *Streptococcus*, *Corynebacterium*, *Akkermansia*, and *Escherichia*, cluster into evolutionarily coherent lineages consistent with human-associated microbiomes. This clustering indicates ecological filtering and selective retention of host-derived microbes in burial contexts [37]. In contrast, control-enriched taxa showed phylogenetic dispersion, consistent with broader environmental adaptation and generalist lifestyles [38].

Interestingly, while *Ruminococcus* is typically a core human gut genus, it was enriched in some control samples. This may reflect detection of environmentally adapted lineages within the *Ruminococcaceae* family, which includes members isolated from soil, plants, and anaerobic ecosystems [39]. Limitations of genus-level 16S classification likely contributed to ambiguous source attribution in this case [40].

Together, these findings affirm that microbial phylogenetics can differentiate between anthropogenically shaped and ambient environmental communities. Our use of phylogenetic overlays incorporating both enrichment and ecological origin supports the utility of phylosymbiosis-based frameworks for postmortem microbial inference [41].

### Microbial clues to diet, environment, and trade

In addition to human-associated microbes, burial soils contained signatures from taxa linked to plant and animal sources. For example, the presence of *Arenimonas oryziterrae*, a rhizobacterium associated with rice cultivation, suggests exposure to domesticated animals and crops, *Clostridium cellobioparum*, a cellulose-degrading bacterium typically found in bovine rumen, and *C. isatidis,* a unique indigo-reducing bacterium isolated from a woad dye [42–44]. These findings offer insight into the foodways, agriculture, and trade practices of the NYABG population in postcolonial New York.

Historical records indicate that rice cultivation was introduced to the Americas through the transatlantic slave trade, with enslaved Africans bringing both the crop and knowledge of its cultivation. While rice was most intensively grown in the Carolinas, it was also circulated through trade to northern colonies, including New York [45]. Similarly, cattle were introduced by European settlers and became essential to colonial agriculture, contributing to milk, meat, and labor in urban areas like New York [46]. While New York was not itself a major producer of indigo due to the limited agricultural success by its Dutch colonists, indigo-dyed materials from southern slave plantations were widely traded through the city, therefore plausible that associated microbial taxa reflect the processing and handling of indigo in New York’s colonial textiles factories [47]. The detection of these microbial taxa, absent from controls, supports their authenticity as historical signals and further validates the burial soil microbiome as a proxy for interpreting lived experience.

### Historical public health and epidemiology

Although species-level resolution was not achieved for pathogens such as *S. dysenteriae*, *Listeria monocytogenes*, or *C. diphtheriae*, the detection of their genera may reflect historical exposure to dysentery, foodborne illness, and diphtheria. These findings are consistent with documented infectious disease burdens in postcolonial New York [48].

Dysentery, then known as “bloody flux,” was rampant on slave ships and common in urban centers with poor sanitation [49]. Diphtheria also emerged as a major public health threat in the 19th century, causing high childhood mortality in New York [50]. Our findings suggest the potential for burial soil metagenomes to contribute to reconstructions of population-level health patterns. Future metagenomic work that employs shotgun sequencing or targeted pathogen capture could enhance taxonomic resolution and provide functional insights into disease ecology.

### Limitations and considerations

This study acknowledges key limitations. Primary among them is the lack of a temporal or archaeological comparator cohort, burial soils from another similarly aged site were not available for comparison. Additionally, while environmental controls were sampled from nearby NYC parks to minimize spatial bias, we cannot fully rule out differences due to collection depth, soil history, composition, or modern microbial inputs. Such contributors could independently shape microbial communities and must be considered as potential confounding factors. Moreover, although our sequencing approach captured broad taxonomic profiles, 16S rRNA gene data alone cannot resolve strain-level variation or functional potential. Additional iterations of this work incorporate shotgun metagenomics to improve taxonomic resolution and confirm burial inhabitant derived human DNA sequences. To address ascertainment bias, current research projects at North Carolina and South Carolina burial sites incorporate co-sampling of human remains (e.g. molars) to assess the resolution of human contributed evidence detected from burial soil.

### Ethical considerations and implications for the NYABG population

The NYABG represents one of the most significant archaeological sites related to the African diaspora in North America. Ethical stewardship of its legacy requires balancing scientific inquiry with respect for descendant communities. Our use of nondestructive soil sampling avoids interference with human remains while recovering meaningful biological data. This approach aligns with calls for community-informed research that respects cultural values and prioritizes equity in scientific discovery [51].

Beyond methodology, our findings illuminate aspects of diet, environment, and health that contribute to understanding systemic inequality, resilience, and everyday life in postcolonial New York. The microbial archive preserved in burial soils represents more than decomposition, it is a living record of social, biological, and environmental history.

Because burial soils represent not just environmental soil but also decomposed tissue and fine bone fragments from the interred individual, their study carries ethical considerations similar to analyses of human remains. It is therefore essential that interpretations, particularly those concerning health, diet, or disease, be communicated transparently with descendant and stakeholder communities, ensuring that these findings are contextualized as hypotheses rather than definitive conclusions.

### Conclusion

In sum, this work redefines what constitutes “human remains” in microbiome science. By demonstrating the long-term preservation of host-specific microbial lineages in soil, we present a new frontier in archaeometagenomics and bioarchaeology. Burial soils are biologically informative, ethically accessible, and temporally stable, making them indispensable for future investigations of ancient human life. As sequencing technologies evolve and ethical frameworks mature, we envision microbial profiling becoming a standard complement to osteological and isotopic methods. Soil microbiomes are not merely the residue of death, but ecological and biological archives of life, offering new pathways for reconstructing history, health, and humanity.

## Data Availability

The soil samples and all associated data underlying this study were provided by the National Park Service (NPS) and the governing body of the General Services Administration (GSA), with consent from community stakeholders, for research purposes. Data will be shared upon reasonable request to the corresponding author and with permission from the National Park Service. All bioinformatic scripts can be found here: https://github.com/The-ASHES-Laboratory/ABG_16S.
